# LVAD with concomitant rapid deployment valve implantation – a case report

**DOI:** 10.1186/s13019-019-0944-5

**Published:** 2019-07-01

**Authors:** Daniel D. Holloway, Lindsay C. Jones, Soo J. Howell, Jonathan D. Rich, Duc Thinh Pham

**Affiliations:** 1 Division of Cardiac Surgery, Center for Advanced Heart Failure, Bluhm Cardiovascular Institute, Northwestern University Feinberg School of Medicine and Northwestern Memorial Hospital, 201 E Huron St, Suite 11-140, Il, Chicago, IL 60611 USA; 2 Division of Cardiology, Northwestern University Feinberg School of Medicine and Northwestern Memorial Hospital, Chicago, IL USA

**Keywords:** Left ventricular assist device, Aortic valve replacement, Rapid deployment valve, Mechanical circulatory support

## Abstract

**Background:**

Aortic valve insufficiency can have significant hemodynamic consequences for patients with left ventricular assist devices. A circulation loop can limit systemic blood flow and increase left ventricular filling pressure.

**Case presentation:**

A 64-year-old male with non-ischemic dilated cardiomyopathy underwent Heartware™ HVAD left ventricular assist device implantation with successful concomitant aortic valve replacement with an Edwards Intuity rapid deployment prosthetic valve.

**Conclusions:**

The use of this rapid deployment valve may have benefits over other techniques including shorter cross clamp times during surgery, intermediate-long term durability, and preservation of aortic valve opening to allow for potential ventricular recovery. The Intuity rapid deployment valve should thus be considered a viable and suitable option for aortic insufficiency intervention during LVAD implantation.

## Background

Aortic valve insufficiency (AI) can have significant hemodynamic consequences for patients with left ventricular assist devices (LVAD). The regurgitation can create a circulation loop limited to the left ventricle (LV), LVAD, and ascending aorta, leading to decreased systemic blood flow and increased LV filling pressure [1, 2]. Consequently, greater than mild AI is generally considered an indication for intervention on the aortic valve (AV) at the time of LVAD implantation [3]. The options for intervention include patch occlusion, AV repair, or replacement with a bioprosthetic valve [1, 4, 5].

### Case presentation

A 64 year old man with non-ischemic dilated cardiomyopathy presented with exertional dyspnea, and progressive New York Heart Association class IV symptoms despite treatment with optimal medical therapy. Following admission to our hospital, the patient was started on a milrinone infusion, but developed refractory ventricular tachycardia associated with worsening cardiogenic shock. This necessitated emergent institution of femoral veno-arterial Extracorporeal Membrane Oxygenation (ECMO). While on ECMO, the patient’s condition stabilized but it was not possible to wean support. It was determined that a durable LVAD would be the appropriate therapy as a bridge to transplant.

Pre-operative echocardiography demonstrated a severely dilated LV with an ejection fraction of 15%. The right ventricle was moderately dilated with moderate dysfunction. The AV was mildly thickened and calcified with moderate AI. Both the mitral and tricuspid valves had severe, functional regurgitation.

The patient underwent AV replacement with a 23 mm Intuity valve (Edwards Lifesciences, Irvine, CA), tricuspid valve repair with a 30 mm MC3 annuloplasty ring (Edwards Lifesciences, Irvine, CA), and HeartWare™ HVAD implant (Medtronic, Minneapolis, MN). The surgery was performed via median sternotomy with aortic and bi-caval cardiopulmonary bypass. The heart was arrested with Del Nido cardioplegia via an antegrade cannula. Through an oblique aortomy the AV cusps were excised and the annulus debrided. The annulus was sized to a 23 mm Intuity valve, and the valve was implanted in standard fashion per the “Instructions for Use”. The aortomy was then closed and the cross-clamp was removed after 51 min. With beating-heart cardiopulmonary bypass, the tricuspid valve was repaired and the LVAD implanted in standard apical-to-ascending aortic fashion. The patient was weaned from cardiopulmonary bypass after 178 min without difficulty.

Post-operatively, the patient was extubated on day 4. He developed a delayed pericardial effusion requiring re-exploration on day 15. He was discharged in stable condition on day 20. Echocardiography prior to discharge demonstrated a well-seated, prosthetic valve with a peak gradient of 3 mmHg and no AI. The AV opened every third beat and the LV was decompressed consistent with a well-functioning LVAD. There was trivial tricuspid and mitral regurgitation.

### Discussion and conclusions

To our knowledge, this case is the first reported use of an Edwards Intuity rapid deployment valve with concomitant LVAD implantation. In this case, the valve was successful in correcting moderate AI with a good short-term result.

With pre-operative moderate AI, this patient had an indication for surgical intervention on the AV. Adding concomitant valve intervention increases the duration and complexity of LVAD implantation; associated with this is an increased risk of mortality [6]. There is equipoise as to which surgical technique is best to address AI. Limited by short follow-up times and relatively low case numbers, the literature is only now beginning to elucidate which techniques should be used for certain patients.

Surgical occlusion and patching of the AV annulus has been performed with good short-term success [1]. However, there is the obvious disadvantage of rendering the patient 100% dependent on LVAD flow. There is also no possibility of bridge- to- recovery. An analysis of the Interagency Registry for Mechanically Assisted Circulatory Support database found that AV occlusion was associated with increased mortality [2]. For this reason we consider this technique to be less desirable.

The central coaptation repair stitch (Park’s stitch) has been shown to be efficacious in decreasing AI in the short term [7–10]. A retrospective assessment at a single center found the two year results of the Park’s stitch is acceptable with decreased AI compared to patients that did not receive AV repair [4]. This technique has advantages of a relatively short cross-clamp time and no significant added expense. However, this technique has limitations as it restricts blood flow through the valve to the lateral aspects of the cusps. This is not an ideal technique for a potential bridge-to-recovery patient. Also, due to the central coaptation, the possibility of later percutaneous intervention on the AV may be technically challenging. Furthermore, after repair, some patients develop progressively worsening AI. Fukuhara et al. found 32% of patients with Park’s stitch had significant AI at two years [10].

Bioprosthetic valves have been proposed as a solution for AI during LVAD implantation [5, 8]. Bioprosthetic AVs offer a reliable way to restore normal valve function and may open intermittently to permit some LV ejection. A further advantage of bioprosthetic valve implantation is if the patient recovers ventricular function, the prosthetic valve would accommodate weaning from the LVAD. A disadvantage of prosthetic valve implantation is it requires a longer cross-clamp and cardiopulmonary bypass time than the other described interventions. On a heart with severely impaired biventricular function, this longer cross-clamp time may be poorly tolerated while trying to wean cardiopulmonary bypass or during the peri-operative period. This is particularly important for patients who have borderline right ventricular function pre-operatively such as the patient in this case.

Rapid deployment valves such as the Edwards Intuity valve are particularly attractive for this patient group with poor cardiac function and little ventricular reserve as they can be implanted with shorter cross-clamp times than a traditional surgical valve [11]. The individual benefit of a decreased cross-clamp time is likely variable and intuitively would depend on the pre and peri-operative stability of the patient. Fundamentally though, reducing cardiac ischemic time without compromising efficacy of the surgery is a worthwhile goal. Borger et al. also demonstrated the Intuity valve has superior hemodynamics compared to traditional stented valves, [11] although in a LVAD patient this benefit is of unclear significance. The disadvantage of the rapid deployment valves is increased cost over a traditional surgical valve, and a significantly greater cost than Park’s stitch. On the other hand, the cost per valve remains much less than a potentially alternative method of rapid valve deployment such as a Transcatheter Aortic Valve Replacement valve. Another potential disadvantage of rapid deployment valves is that the balloon expandable frame exerts radial force on the LV outflow tract which may increase the rate of permanent pacemaker implantation [12].

In conclusion, a rapid deployment Intuity valve was successfully implanted to treat AI on a patient undergoing concomitant LVAD implantation. This produced a good short-term result with ongoing follow-up pending. We believe the Intuity valve is a reasonable option for addressing AI at the time of LVAD implantationas it offers the reliable function of a prosthetic valve with a relatively short cross-clamp time.

## Data Availability

Please contact author for data requests.

## Abbreviations

AI: Aortic valve insufficiency
AV: Aortic valve
ECMO: Extracorporeal membrane oxygenation
LVAD: Left ventricular assist device
LV: Left ventricle
